# The Value of Fetuin-A as a Predictor to Identify Takotsubo Patients at Risk of Cardiovascular Events

**DOI:** 10.3390/jcdd8100127

**Published:** 2021-10-05

**Authors:** Albert Topf, Moritz Mirna, Nina Bacher, Vera Paar, Christoph Edlinger, Lukas J. Motloch, Sarah Gharibeh, Marwin Bannehr, Uta C. Hoppe, Michael Lichtenauer

**Affiliations:** 1 Department of Internal Medicine II, Paracelsus Medical University, 5020 Salzburg, Austria; a.topf@salk.at (A.T.); m.mirna@salk.at (M.M.); n.bacher@salk.at (N.B.); v.paar@salk.at (V.P.); l.motloch@salk.at (L.J.M.); s.gharibeh@salk.at (S.G.); u.hoppe@salk.at (U.C.H.); 2 Department of Cardiology, Heart Center Brandenburg, 16321 Bernau bei Berlin, Germany; christophroland.edlinger@immanuelalbertinen.de (C.E.); marwin.bannehr@immanuelalbertinen.de (M.B.)

**Keywords:** fetuin-A, takotsubo cardiomyopathy, cardiovascular events

## Abstract

Introduction: Takotsubo cardiomyopathy (TTC) remains a life-threatening disease with the risk of decompensated heart failure and arrhythmias. Valid markers for the prediction of outcome are unavailable. The novel biomarkers fetuin-A, matrix metalloproteinases-2 (MMP-2), myeloperoxidase (MPO), Syndecan-1 and CD40-L show promising results for risk stratification of cardiovascular patients. Nevertheless, clinical implementation has not been investigated in TTC patients. Methods: To investigate this issue, we evaluated clinical complications in 51 patients hospitalized for TTC and measured the serum levels of fetuin-A, MPO, MMP-2, Syndecan-1 and CD40-L within 24 h after admission. Results: Serum levels of Fetuin-A correlated inversely with the risk of cardiac decompensation and all cause complications within the acute phase of TTC. Fetuin-A levels over 190.1 µg/mL (AUC: 0.738, sensitivity 87.5%, specificity: 52.6%) indicate an acute phase of TTC without cardiac decompensation. Despite lower fetuin-A levels in patients with all cause complications, the combined endpoint remained slightly unmet (*p* = 0.058, AUC: 0.655). Patients with fetuin-A levels over 213.3 µg/mL are at risk of experiencing hemodynamic relevant rhythm disorders (AUC: 0.794; sensitivity: 75.0%, specificity: 79.1%). Other biomarkers failed to reveal a prognostic impact. Pro-BNP and hs troponin levels at admission did not predict adverse cardiac events. Conclusion: Fetuin-A is a promising marker in our study and could be of benefit for the prediction of short-term adverse cardiac events in TTC patients. Therefore, fetuin-A might be of value to evaluate an individual’s risk for complications within the acute phase of TTC and to individually choose the time of intensive care and hospitalization.

## 1. Introduction

Takotsubo cardiomyopathy (TTC) is a condition characterized by acute left ventricular dysfunction with symptoms similar to an acute myocardial infarction, but in the absence of a significant coronary stenosis [1]. Despite, an incidence of 7.5% in the female population, 3% of all suspected acute coronary syndromes (ACSs) are caused by TTC [2]. The syndrome is often triggered by emotional and physical stress factors and comprises reversible wall motion abnormalities involving apical, midventricular or basal segments of the left ventricle [3].

In general, TTC is found in postmenopausal women and is triggered by emotional or physical stressors. The pathogenic mechanism leading to this disease hasn’t been elucidated, but it has been suggested that the heart muscle inadequately responds to excessive catecholamine release [4].

Patients with TTC usually have a good prognosis and almost perfect recovery, with resolution of wall motion abnormalities within a few days in 96% of the cases [5]. Despite this, TTC remains a life-threatening disease in the acute phase, with a mortality rate of one to two percent and 20% develop congestive heart failure necessitating a forced intravenous diuretic therapy or even ventilation therapy. Life-threatening ventricular arrhythmias occur in 8.6% of TTC patients. Left ventricular wall rupture or thrombosis and cardiogenic shock are also reported [6]. Valid parameters indicating adverse cardiac events and predicting the general prognosis are unknown or rather not extensively investigated.

In contrast to TTC, in ACS the occurrence of complications is better studied, due to the higher prevalence in the population. Therefore, the needed time for intensive care and for hospitalization is more predictable. The aim of this study is to investigate the predictive value of novel biomarkers to estimate the risk of adverse cardiac events in TTC patients and therefore to evaluate an individual’s risk for complications within the acute phase of the disease. Besides the clinical status, it might be helpful to choose the necessary time of intensive care and hospitalization by analyzing additional biomarkers.

We investigated the clinical use of fetuin-A for the prediction of adverse cardiac events in TTC patients. This marker has already been studied in cardiovascular diseases including heart failure and acute coronary syndrome. Fetuin-A is a phosphorylated glycoprotein and a member of the fetuin group of serum-binding proteins, which are all comprised of three O-linked and two N-linked oligosaccharide chains. Fetuin-A counteracts proinflammatory cytokine production [7]. Fetuin-A is mainly synthesized by hepatocytes but may also be produced in other organs, such as the kidneys and the tongue. Serving as a mediating signal for antagonizing growth factor, fetuin-A inhibits the mineralization of the skeletal matrix. Fetuin-A can also bind with cationic ions like calcium, indicating that it is capable of inhibiting ectopic calcification. Studies have shown that low fetuin-A concentration is associated with cardiovascular death and may play an important role in the prognosis of patients with ACS. Decreased serum fetuin-A concentrations may affect cardiac functions by increasing cardiac fibrosis and calcification, and thus promote cardiovascular disease progression [8]. Considering the prognostic impact of this marker in patients’ acute coronary syndrome or heart failure, low fetuin-A levels have been found to be associated with infarct size, left ventricular function, all-cause mortality and the extent of systolic heart failure [9]. It is suspected that abnormalities in the Ca2^+^-regulating system may be involved in the pathology of TTC. Vitamin D insufficiency has already been described in a study of 25 TTC patients by Dande et al. [10]. This suspicion is confirmed by the fact that there is a seasonal variation in the occurrence of TTC [11]. Furthermore, in gene expression analysis from endomyocardial biopsies of TTC patients, Holger et al. showed abnormalities of calcium handling proteins [12]. Therefore, fetuin-A, as a calcium-dependent biomarker, might have a predictive value in TTC.

Matrix metalloproteinases (MMPs) are a family of proteolytic enzymes, which are modified by inflammatory signals in order to mediate alterations in the extracellular matrix. Members of the MMP family play a role in inflammatory processes and chronically mediate tissue remodeling. MMPs are crucial in vascular remodeling, most importantly in advancing atherosclerotic plaque formation. MMP activation modifies the composition of the plaque and may be partly responsible in the process of plaque rupture. MMPs also play a role in cardiac remodeling after myocardial infarction and in the development of dilated cardiomyopathy. Soluble MMPs are potential predictors for plaque rupture and coronary risk [13]. Higher levels of MMPs are associated with cardiovascular death and with all-cause mortality [14]. In a small study of Essa et al., MMPs profiles of TTC patients were similar to those described in hypertensive heart disease and diastolic heart failure and different than the profiles following myocardial infarction [15]. MMPs were reported in a small study from Parkkonen et al. to better differentiate TTC from ACS than troponin T. In TTC, low MMP levels may reflect decreased proteolysis and therefore increased transient fibrosis, perhaps in part explaining the left-ventricle impairment [16].

Myeloperoxidase (MPO) is a member of the superfamily of heme peroxidases that is mainly expressed in neutrophils and monocytes. MPO-derived reactive species play a key role in neutrophil antimicrobial activity and human defense against various pathogens primarily by participating in phagocytosis. Elevated MPO levels in circulation are associated with inflammation and increased oxidative stress [17]. There has been an association between MPO levels and various cardiovascular diseases, including coronary artery disease, congestive heart failure, arterial hypertension, pulmonary arterial hypertension, peripheral arterial disease, myocardial, stroke, cardiac arrhythmia and venous thrombosis. Increased MPO levels indicate a poor prognosis, because of a higher risk of all-cause and cardiovascular-related mortality [18]. An increased production of reactive oxygen species (ROS) is suspected to be involved in the pathophysiological process of TTC. ROS may cause transient coronary and peripheral endothelial dysfunction leading to a transient impairment of myocardial contraction due to stunning. This suspicion derives from the analysis of animal and human endomyocardial biopsies [19]. Endogenous hydrogen sulfide (H2S) was reported by Zhang et al. to be protective in myocardial dysfunction in TTC rats and could therefore be a potential therapeutic target for alleviating β-adrenergic system overstimulation-induced cardiovascular dysfunction [20]. Therefore, MPO, as one of the best studied markers for ROS-induced cardiovascular damage, seems promising to analyze for its predictive value in TTC.

Syndecan-1 is a transmembrane proteoglycan that exerts its functions mainly via its heparan sulfate chains. Syndecan-1 is associated with the development of cardiac fibrosis and atherogenesis. Syndecan-1 participates in regenerative cardiovascular processes and is therefore associated with tissue injury and inflammatory processes [21]. This complex molecule is also involved in the lipid metabolism, by altering the clearance of cholesterol particles. Syndecan-1 is thought to reveal prognostic aspects in patients with heart failure [22]. Epicardial and/or microvascular coronary artery spasm is one suspected pathogenetic mechanism for TTC. Endothelial dysfunction, a pathological state of the endothelium characterized by an imbalance between vasoconstricting and vasodilating factors, may be responsible for myocardial stunning. Therefore, Syndecan-1, as a marker of tissue injury and inflammatory processes might have predictive aspects in TTC [4].

The CD40─CD40L pathway plays a role in the prothrombotic and the proinflammatory system. CD40 and its ligand were first discovered on the surface of activated T cells, but its presence on B cells, antigen-presenting cells, mast cells and finally platelets has already been confirmed [23]. CD40L contributes to atherosclerosis and atherothrombosis, and therefore, CD40L is suspected to trigger acute coronary syndromes. The blockade of this pathway with anti-CD40L antibodies may prevent or delay the progression of atherosclerosis. Levels of CD40L are reported to predict clinical outcomes in patients with acute coronary syndromes [24]. Inflammatory processes, characterized by myocardial macrophage inflammatory infiltrates, changes in the distribution of monocyte subsets and an increase in systemic proinflammatory cytokines, are suspected to be involved in the pathophysiological process of TTC. Many of these changes might persist for at least 5 months, suggesting a low-grade chronic inflammatory state [25]. The process may be initiated by a break in immunological self-tolerance. Tolerance to most self-antigens is ensured through a T-cell maturation step in the thymus. However, the process of inducing central tolerance is missing for cardiac myosin. T-cells with the potential to react to cardiac myosin are present in healthy individuals and easily activated if cardiac myosin is encountered under inflammatory conditions [26]. As a result, CD40, as a T-cell-mediated cardiovascular marker, was chosen to be analyzed for its predictive value in TTC.

## 2. Methods

### 2.1. Patients and Controls

The study was approved by the local ethics committee (415-E/2230/10-2018) and was performed in accordance with the Declaration of Helsinki and Good Clinical Practice. All patients provided written informed consent prior to enrollment.

In this prospective study, we recruited 51 patients with TTC, who were treated at the University Hospital of Salzburg. They were enrolled if they fulfilled the Mayo Clinic Diagnostic Criteria for TTC [27]. Serum samples were collected within 24 h after the admission to hospital. Data on clinical presentation, precipitating factors, cardiovascular risk factors, medications and demographics were obtained as well. All patients received at least 24 h of ECG monitoring at an intensive ward and the majority had a 24 h ECG in the following hospitalization. Documented arrhythmia, necessitating immediate pharmacological therapy or electrical cardioversion were implemented in this study. Clinical and radiographic signs of fluid retention were incorporated as signs of cardiac decompensation, requiring forced diuresis and/or ventilation therapy. Besides the endpoint of hemodynamic compromising supra- and ventricular arrythmias and the endpoint of clinical and/or cardiac decompensation, a third endpoint of all adverse cardiac events was defined. The third endpoint of all-cause complications encompasses all TTC patients with cardiac decompensation and/or arrythmias and/or cardiogenic shock and/or left ventricular wall rupture or thrombosis and/or cardiovascular death within the acute phase of TTC.

### 2.2. Blood Samples

Blood samples were collected from a cubital vein using a clean vein puncture under controlled venous stasis. The collection tubes were centrifuged within 20 min after blood withdrawal and the obtained plasma samples were frozen at −80 °C until further measurements were conducted. Routine blood analysis was performed immediately after blood withdrawal according to clinical standards.

### 2.3. Biomarker Analysis

Serum levels were analyzed at baseline. Serum levels of fetuin-A, MPO, MMP-2, Syndecan-1 and CD40-L were measured by using commercially available enzyme linked immunosorbent assay (ELISA) kits (DuoSet ELISA, DY523B, R&D Systems, Minneapolis, MN, USA). ELISA assays were performed in accordance with instructions supplied by the manufacturer. In short, serum samples and standard proteins were added to the multiwell plate coated with the respective capture antibody and incubated for 2 hours. Plates were then washed using washing buffer (Tween 20, Sigma Aldrich, St. Louis, MO, USA) and phosphate buffered saline solutions. In the next step, a biotin-labelled antibody was added to each well and incubated for another 2 h. ELISA plates were washed another time and a streptavidin-horseradish-peroxidase solution was added. After adding tetramethylbenzidine (TMB; Sigma Aldrich, St. Louis, MO, USA), a color reaction was achieved. Optical density was measured at 450 nm on an ELISA plate reader (iMark Microplate Absorbance Reader, Bio-Rad Laboratories, Vienna, Austria).

### 2.4. Statistical Analysis

Statistical analysis was performed using SPSS (22.0, SPSS Inc., New York, NY, USA). The Kolmogorov─Smirnov test was used to assess the distribution of data in the study population. As most parameters and biomarker concentrations were not normally distributed, all values were given as median and interquartile range (IQR). Median values between groups were compared by Kruskal─Wallis test with Dunn’s post hoc test. Correlation analysis was performed using Spearman’s rank-correlation coefficient. ROC analysis was performed and an optimal cutoff was calculated by means of the Youden index. Areas under the curve (AUC) were compared as described by Hanley and McNeil [28]. A *p* < 0.05 was considered statistically significant.

## 3. Results

### 3.1. Baseline Characteristics

Baseline characteristics of TTC patients are shown in Table 1. TTC patients had a median age of 74.0 years (IQR: 62.0–78.0 years). Left ventricular ejection fraction of patients at admission was reduced (median: 40.0%, IQR: 199.7–223.6%). BNP and hs troponin levels were elevated. Regarding comorbidities, hypertension was the most prevalent in TTC patients. Cardiac decompensation with either radiographic and/or clinical signs of fluid retention were found in 19 out of 51 patients. Eight patients had hemodynamic compromising ventricular and supraventricular arrythmias. Considering all complications together, in 23 out of 51 TTC patients, adverse cardiac events were observed. There were no fatal incidents among the patients included in this study. 

None of the included patients had a history of significant coronary artery disease or of heart failure. 21 out of 51 patients didn’t have a coronary artery disease in the acute coronary angiography and the rest had nonsignificant coronary artery stenosis. 11 out of 51 patients had preceding emotional triggers.

### 3.2. Correlation of Biomarkers with Clinical Characteristics

Correlations between fetuin-A, MPO, MMP-2, Syndecan-1, CD40-L and patient characteristics are depicted in Table 2. Correlation analysis revealed a correlation of arrhythmia with serum levels of fetuin-A in TTC patients (rs: 0.370, *p* = 0.008) and an inverse correlation with the risk of cardiac decompensation during the acute phase of TTC (rs: −0.400, *p* = 0.004). There was no correlation of serum levels of fetuin-A of TTC patients with clinical characteristics. Considering other biomarkers, there was no correlation with endpoints of adverse cardiac events.

### 3.3. Biomarkers as Indicators of Cardiac Decompensation within the Acute Phase of TTC

**Figure 1:**
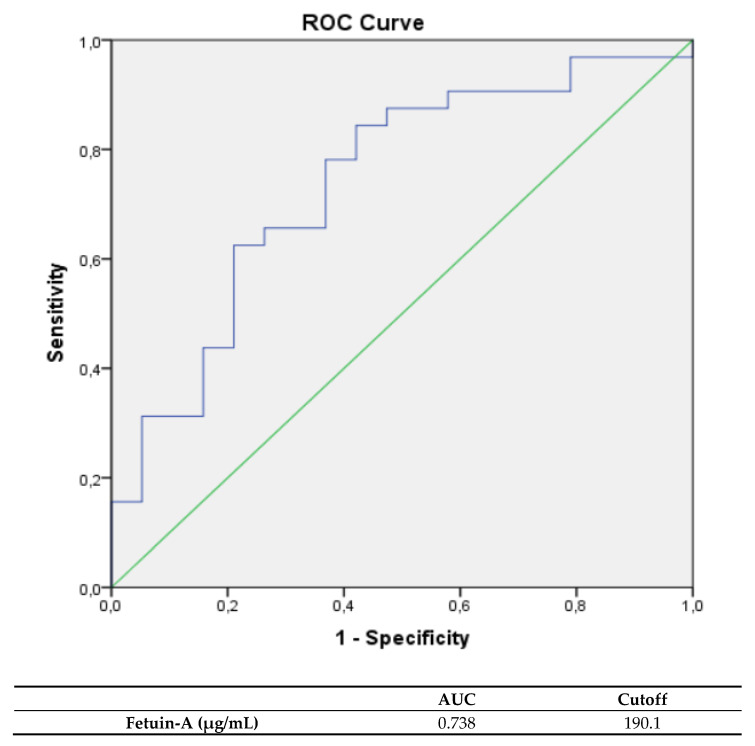
ROC curves and cutoff scores for fetuin-A for the prediction of a clinical course with cardiac decompensation in the total cohort.

Fetuin-A levels of patients (median: 190.0 µg/mL, IQR: 177.8–203.7 µg/mL), suffering a cardiac decompensation, significantly differed from patients with a euvolemic form (median: 208.2 µg/mL, IQR: 199.7–223.6 µg/mL, *p*: 0.005). Fetuin-A correlated inversely with the risk of cardiac decompensation within the acute phase of TTC. Therefore, low fetuin-A levels indicate a risk of cardiac decompensation. Fetuin-A levels in TTC patients of 190.1 µg/mL (sensitivity: 87.5%, specificity: 52.6%, PPV: 60.0%, NPV: 87.5%) were determined to predict a clinical course with fluid retention within the acute phase (see Figure 1). Other biomarkers failed to predict the risk of cardiac decompensation within the acute phase of TTC and didn’t significantly differ among the two subgroups. When considering classic biomarkers, Pro-BNP ((median: 4299.5 pg/mL, IQR: 763.3–5816.8 pg/mL) vs. (median: 2680.5 pg/mL, IQR: 420.4–4373.5 pg/mL); *p*: 0.216) and hs troponin ((median: 261.0 pg/ml, IQR: 51.0–837.0 pg/mL) vs. (median: 139.0 pg/mL, IQR: 53.0–287.3 pg/mL); *p*: 0.209) levels at admission were nonsignificantly higher in TTC patients with following cardiac decompensation within the acute phase. 

When considering patients’ clinical characteristics, left ventricular ejection fraction of patients without fluid retention (median: 45%, IQR: 40.0%–50.0%) was significantly higher compared to patients with cardiac decompensation (median: 40%, IQR:35.0%–45.0%; *p* = 0.005). Patients with cardiac decompensation were significantly older (median: 78.0 years, IQR: 65.0–81.0 years vs. median: 66.0 years, IQR: 59.3.0–75.8 years; *p* = 0.006). Sex was not a determinant for the risk of cardiac decompensation.

### 3.4. Biomarkers as Predictors for Arrhythmia within the Acute Phase of TTC

**Figure 2:**
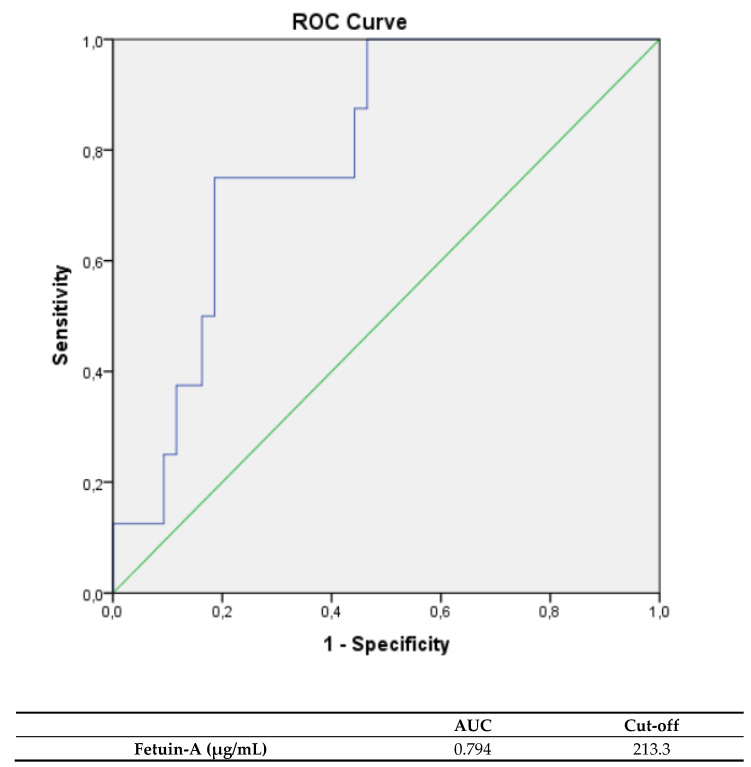
ROC curves and cutoff scores for fetuin-A for prediction of arrhythmic events in the total cohort.

Fetuin-A levels were significantly higher in TTC patients suffering an arrhythmia during hospitalization, compared to TTC patients without arrythmias (median: 217.2 µg/mL, IQR: 206.4–226.1 µg/mL vs. median: 200.6 µg/mL, IQR: 187.8–211.3 µg/mL; *p* = 0.007). Fetuin-A levels showed a correlation with arrythmias in the acute phase of TTC (rs: 0.370, *p* = 0.008) A ROC analysis was performed and the AUC was calculated for fetuin-A levels, indicating arrhythmia in TTC patients (see Figure 2). The calculated cutoff value for an outcome, complicated by arrhythmia, was 213.3 µg/ml (AUC: 0.794; sensitivity: 75.0%; specificity: 79.1%; PPV: 75.0%; NPV: 79.1%). Other biomarkers failed to prognose arrhythmia in TTC patients. Pro-BNP ((median: 2652.0 pg/ml, IQR: 785.0–6071.0 pg/mL) vs. (median: 3631.0 pg/mL, IQR: 538.4–4761.0 pg/mL); *p*: 0.939)) and hs troponin ((median: 82.5 pg/mL, IQR: 39.5–549.0 pg/mL) vs. (median: 186.0 pg/mL, IQR: 69.0–395.0 pg/mL); *p*: 0.422) levels at admission were nonsignificantly lower in TTC patients with following arrythmias within the acute phase. Furthermore, there was no significant difference of baseline characteristics, including sex, between patients suffering an arrhythmia and patients without rhythm events in the acute phase of TTC.

### 3.5. Biomarkers, as Indicators for All Cause Complications

Fetuin-A levels of TTC patients with all cause complications (median: 195.0 µg/mL, IQR: 185.1–216.0 µg/mL) were lower compared to those of TTC patients without all cause complications (median: 207.3 µg/mL, IQR: 195.5–223.6 µg/mL). Fetuin-A levels showed an inverse correlation with all cause complications in the acute phase of TTC (rs: −0.268, *p* = 0.058). A ROC analysis was performed and the AUC was calculated for fetuin-A levels, indicating all-cause complications in TTC patients. The calculated cutoff value for an outcome without all-cause complications was 204.6 µg/mL (AUC: 0.655; sensitivity: 60.7%; specificity: 69.6%). Other biomarkers showed less accuracy in predicting all-cause complications. When considering classic biomarkers, Pro-BNP ((median: 3601.5 pg/mL, IQR: 1108.3–5816.8 pg/mL) vs. (median: 2654.0 pg/mL, IQR: 366.8–4373.5 pg/mL); *p*: 0.159)) and hs troponin ((median: 225.0 pg/mL, IQR: 53.0–837.0 pg/mL) vs. (median: 139.0 pg/mL, IQR: 50.8–287.3 pg/mL); *p*: 0.161) levels at admission were non-significantly higher in TTC patients with following all-cause complications within the acute phase. Sex was not a determinant for all-cause complications.

## 4. Discussion

Takotsubo cardiomyopathy is an acute reversible heart failure condition with functional recovery within a few weeks in almost 96% of cases. Despite a good general prognosis and an often-attributed benign pathology to its self-limiting course, life threatening complications and even fatal incidents may worsen clinical prognosis [29]. Therefore, monitoring on an intermediate care unit is standard clinical practice in the acute phase of TTC [30]. Fluid retention, hemodynamic compromising arrhythmias or even cardiogenic shock may require a transfer to an intensive care ward for a ventilation therapy or even extracorporeal circulation therapy.

TTC mimics an ACS and therefore, predictors for the outcome are often adopted from recommendations of ACS. Due to the higher prevalence of ACS, this approach seems appealing, but a different pathophysiological background may render this concept futile [31]. In ACS, scores such as the GRACE score, the PAMI-II criteria or the Zwolle primary PCI Index, are validated to predict the outcome and to guide the duration of intensive care or the time of hospitalization [32]. However, large-scale studies or guidelines to plan the needed time of intensive care or the duration of hospitalization of TTC patients are unavailable.

In an analysis from the Spanish National Registry on TTC, physical triggers, including infections, surgical procedures, physical activities, severe hypoxia and neurological events were identified as the worst predictors of short-term prognosis. Age > 70 years, diabetes mellitus, left ventricular ejection fraction < 30% and shock at admission were considered to affect long-term prognosis [33]. The adoption of the GRACE-risk score for the outcome of TTC patients seemed promising in the prediction of fatal incidents in a study including 561 TTC patients. However, specific indicators for the prediction of short-term adverse cardiac events are unavailable. 

Santoro et al. described CEA and CA19-9 to be associated with higher long-term risk of in-hospital MACE and death [34]. Neutrophil/lymphocyte ratio was reported in 160 TTC patients to predict in-hospital complications (sensitivity of 82% and specificity of 58%, AUC: 0.73) and correlated with hospital stay duration [35]. Furthermore, increased IL-6 and IL-10 were associated with higher rates of adverse cardiac events at follow-up [36].

Baseline hs troponin levels in patients with coronary artery disease were associated with clinical outcomes after PCI. Additionally, preprocedural hs troponin levels above the 99th percentile upper reference limit were related to all-cause mortality within 1 year after PCI [37]. In patients with acute coronary syndrome, higher plasma levels of galectin-3 levels were found to be associated with poor survival rate [38]. Furthermore, high FGF-23 plasma levels have been shown to predict mortality and cardiovascular events [39,40], hs troponin and in further biomarker analysis, Galectin-3 didn’t have a prognostic relevance in TTC. Galectin-3 was neither predictive for cardiac decompensation (*rs*: −0.152; *p*: 0.293), for arrhythmias (*rs*: −0.002; *p*: 0.990) nor all-cause complications (*rs*: −0.177, *p*: 0.219).

Among the investigated markers, fetuin-A was the paramount biomarker for the prediction of adverse cardiac events within the acute phase of TTC. Other biomarkers, including MPO, MMP-2, syndecan-1 and CD40-L failed to prognose cardiac decompensation, rhythm disorders or all-cause complications within the acute phase of TTC. 

Fetuin-A levels of patients, suffering a cardiac decompensation and necessitating a forced diuresis and/or ventilation therapy, significantly differed from patients with a euvolemic form. Therefore, patients with low fetuin-A levels under 190.1 µg/ml are at risk of cardiac decompensation. The following results are in accordance with previous trials, investigating the role of fetuin-A in patients with ACS or heart failure. Low fetuin-A levels have been shown to be associated with infarct size, left ventricular function, all-cause mortality and the extent of systolic heart failure [9]. Therefore, low fetuin-A levels might predominantly reflect the risk of cardiac decompensation by de novo diagnosed heart failure. It remains unclear how left ventricular outflow tract obstruction (LVOT), with a described prevalence of approximately 25% of all TTC patients, has an influence on the risk of cardiac decompensation [41]. 

In our study, fetuin-A was showed to be predictive for the identification of patients suffering an arrhythmia with the need of an urgent electrical or pharmacological cardioversion. Fetuin-A levels were significantly higher in TTC patients, experiencing an arrhythmia compared to TTC patients without rhythm disorders. Arrhythmia should be expected in TTC patients with a fetuin-A level over 213.3 µg/mL. The pathophysiological background of the increase of fetuin-A levels in TTC patients experiencing arrhythmia is difficult to interpret and might even be an unexpected finding, as the role of fetuin-A has so far not been investigated for the prediction of arrhythmia and quantifiable predictors for the occurrence of arrythmias have not been identified. Triggers for arrythmias in TTC seem to have a heterogenous origin and therefore, simple predictors to select the optimal duration of cardiac monitoring, used to identify patients at risk of sudden cardiac death (SCD), are not available [42]. The underlying mechanisms of ventricular arrythmias in TTC seem to involve multiple factors including extensive myocardial edema, repolarization disturbances, catecholamines and sympathetic overdrive. In view of the presumed causal association of arrythmia with the onset of TTC, catecholamines and sympathetic activity seem to be particularly important factors in very early ventricular arrhythmic events. In contrast, structural myocardial alterations and repolarization disturbances evolve over several hours/days and seem to play a major role during this period [43]. Additionally, in a retrospective study of 6837 patients, a higher frequency of arrythmias was observed in women than men [44]. In an analysis of 214 TTC patients, newly diagnosed atrial arrythmia were more frequently observed in TTC patients presenting with bigger myocardial damage and lower LVEF [45]. In a recent smaller study of 93 TTC patients, subacute ventricular arrythmias were associated with New York Heart Association (NYHA) class III–IV on admission and higher QTc intervals at 48 hours [46]. Nevertheless, the association of fetuin-A with arrythmias might present new and interesting insights into the occurrence of arrythmias in TTC. In a suspected multifactorial genesis, a correlation with a calcium dependent biomarker such as fetuin-A seems plausible as calcium is one of the main electrolytes being involved in electrophysiological processes and the occurrence of arrhythmias [47]. Additionally, the modification of calcium dependent proteins and the vulnerability of persons with genetic modifications in calcium regulating genes may endorse the speculations of calcium-dependent pathways being specifically involved in the occurrence of arrythmias in TTC patients, and may confirm the role of fetuin-A for the prediction of arrhythmias [48]. The inverse performance of fetuin-A for the prediction of arrythmias compared to the prediction of decompensated heart failure may therefore reflect that different factors are involved in the pathophysiological process. Conversely, low fetuin-A levels for the prediction of the risk of cardiac decompensation predominantly refer to the new onset of heart failure. Left ventricular ejection fraction and heart failure seem to be a less-driving pathogenicity factor for the occurrence of arrythmia in the acute phase of TTC. High fetuin-A levels for the prediction of arrythmias may present new insights into the multivariable genesis of arrythmias by the identification of possible, specific calcium-dependent pathways in TTC or by the identification of TTC patients with sympathetic overdrive, structural myocardial alterations or repolarization disturbances at risk of arrythmias. Biomarkers to quantify the risk of arrythmias have so far not been described, and therefore the results for fetuin-A seem promising. To confirm the role of fetuin-A as a predictor of arrythmias in TTC, large scale studies are necessary.

The goal of determining the value of fetuin-A for the prediction of the combined endpoint of all-cause complication remained slightly unmet. Fetuin-A levels of TTC patients with all-cause complications were lower compared to TTC patients without side effects, but there was no significant difference (*p* = 0.058).

Fetuin-A in combination with scores such as the Grace-risk score, might be of interest to better predict the needed time of monitoring on an intensive ward or the duration of hospitalization. 

## 5. Conclusions

Fetuin-A is a promising biomarker in our study and could be of benefit for the prediction of adverse cardiac events in TTC patients. Therefore, fetuin-A might be of value for physicians to evaluate the needed duration of intensive care or the time of hospitalization of TTC patients.

## 6. Limitations

Among the limitations of the present study is the small cohort. Large-scale studies are required to confirm the results of the present study.

## Figures and Tables

**Table 1:** Baseline characteristics of all TTC patients, given as median and IQR.

	TTC	
	Median	IQR
Age (years)	74.0	62.0–78.0
BMI (kg/m^2^)	24.7	21.8–29.2
EF (%)	40.0	35.0–46.0
Creatinine (µmol/L)	64.2	59.8–79.2
LDL (mg/dL)	90.0	75.0–122.0
CRP (mg/L)	0.4	0.2–0.9
HbA1c (%)	5.4	5.2–5.8
QTc (ms)	458.0	439–491
Heart rate (bpm)	78.0	71.0–90.0
(hs) Troponin (pg/mL)	162.0	53.0-395.0
Pro-BNP (pg/mL)	2866.0	664.6–4919.8
Fetuin-A (µg/mL)	203.2	189.3–216.5
MMP-2 (ng/mL)	111.1	93.9–127.5
MPO (pg/mL)	408461.5	189870.6–840546.0
Syndecan-1 (pg/mL)	598.3	387.5–911.3
CD40-L (pg/mL)	397.7	290.1–784.4
Arrhythmia (*n*)	8/51	
Cardiac decompensation (*n*)	19/51	
All cause complications (*n*)	23/51	
Smoking	15/51 (29.4%)	
Hypertension	38/51 (74.5%)	
Sex (female)	48/51 (94.1%)	

**Table 2:** Bivariate correlation and point-biserial correlation analysis of baseline characteristics and biomarkers.

	Fetuin-A		CD40L		MMP-2		Syndecan-1		MPO	
	*rs*	*p*	*rs*	*p*	*rs*	*p*	*rs*	*p*	*rs*	*p*
Age (y)	−0.215	0.130	−0.095	0.506	0.243	0.086	0.159	0.264	0.120	0.367
BMI (kg/m^2)	0.049	0.734	−0.197	0.165	-0.168	0.238	0.065	0.650	−0.032	0.826
EF (%)	−0.091	0.524	0.053	0.711	0.119	0.405	−0.116	0.416	0.113	0.432
Heart rate (bpm)	−0.099	0.8	−0.039	0.786	-0.177	0.214	0.091	0.527	−0.157	0.272
QTc (ms)	0.183	0.200	−0.121	0.397	0.260	0.065	0.135	0.343	−0.103	0.471
Creatinine (µmol/L)	0.141	0.325	−0.272	0.054	0.349	0.012	0.273	0.053	−0.255	0.112
CRP (mg/dL)	−0.012	0.931	−0.299	0.033	0.198	0.165	0.061	0.671	−0.141	0.322
LDL (mg/dL)	−0.040	0.790	0.070	0.641	-0.271	0.065	−0.027	0.855	0.032	0.833
HbA1c (%)	0.052	0.771	−0.284	0.104	0.382	0.026	−0.056	0.753	−0.307	0.077
Cardiac decompensation (*n*)	−0.400	0.004	−0.047	0.744	0.077	0.591	0.141	0.325	0.095	0.507
Arrhythmia (*n*)	0.370	0.008	−0.013	0.929	0.011	0.939	−0.137	0.336	0.440	0.759
All-cause complications (*n*)	−0.268	0.058	−0.060	0.675	0.040	0.780	0.055	0.702	0.007	0.963

## Data Availability

Available from corresponding author upon reasonable request.
